# RETRACTION: *Persea declinata* (Bl.) Kosterm Bark Crude Extract Induces Apoptosis in MCF‐7 Cells via G_0_/G_1_ Cell Cycle Arrest, Bcl‐2/Bax/Bcl‐xl Signaling Pathways, and ROS Generation

**DOI:** 10.1155/ecam/9806495

**Published:** 2026-10-16

**Authors:** Evidence-Based Complementary and Alternative Medicine

RETRACTION: P. Narrima, M. Paydar, C. Y. Looi, et al., “*Persea declinata* (Bl.) Kosterm Bark Crude Extract Induces Apoptosis in MCF‐7 Cells via G_0_/G_1_ Cell Cycle Arrest, Bcl‐2/Bax/Bcl‐xl Signaling Pathways, and ROS Generation,” *Evidence-Based Complementary and Alternative Medicine*, 2014, 248103, https://doi.org/10.1155/2014/248103.

The above article, published online on 07 April 2014 in Wiley Online Library (https://onlinelibrary.wiley.com/), has been retracted by John Wiley & Sons Ltd. The retraction has been agreed due to concerns identified with the Figures, reported both directly to the publisher and on PubPeer [1]. Further concerns were then identified during the subsequent investigation by the publisher. A summary of the issues are below:

• In Figure 8, the PDM (25 µg/mL) + TNF‐*α* panels appear highly similar to the untreated +1 ng/mL TNF‐*α* panels in Figure 9 of [2], which shares one author
• In Figure 4a, the doxorubicin 2.5 µg/mL panels appear identical to the MCF‐7/doxorubicin panels in Figure 4a of a publication with multiple shared authors [3].
• In Figure 5a, the doxorubicin 2.5 µg/mL panels appear identical to the MCF‐7/doxorubicin panels in Figure 5a of [3].

The authors provided a response and original images. Whilst the overlapping publications [2, 3] were published after this article, the authors did not provide a satisfactory explanation as to why in Figure 8 identical images were used to present different experimental conditions.

After a full assessment, the publisher has lost confidence in the results and conclusions presented. Therefore, the article is retracted.

The authors disagree with the retraction.

## References

[1] belfragei A. , *Persea declinata* (Bl.) Kosterm Bark Crude Extract Induces Apoptosis in MCF-7 Cells via G_0_/G_1_ Cell Cycle Arrest, Bcl-2/Bax/Bcl-xl Signaling Pathways, and ROS Generation, PubPeer. (2025) https://pubpeer.com/publications/B0E71F4B410379C6C64A9ACFB3CCC0. 10.1155/2014/248103 PMC3997877 24808916

[2] Moghadamtousi S. Z. , Kadir H. A. , Paydar M. , Rouhollahi E. , and Karimian H. , *Annona muricata* Leaves Induced Apoptosis in A549 Cells Through Mitochondrial-Mediated Pathway and Involvement of NF-κB, BMC Complementary and Alternative Medicine. (2014) 14, no. 1, 10.1186/1472-6882-14-299. PMC4246449 25127718

[3] Taha H. , Looi C. Y. , Arya A. et al., (6E,10E) Isopolycerasoidol and (6E,10E) Isopolycerasoidol Methyl Ester, Prenylated Benzopyran Derivatives From *Pseuduvaria monticola* Induce Mitochondrial-Mediated Apoptosis in Human Breast Adenocarcinoma Cells, PLoS One. (2015) 10, no. 5, 10.1371/journal.pone.0126126. PMC4422716 25946039

